# Recognizing ancient papyri by a combination of spectroscopic, diffractional and chromatographic analytical tools

**DOI:** 10.1038/srep46236

**Published:** 2017-04-06

**Authors:** J. Łojewska, I. Rabin, D. Pawcenis, J. Bagniuk, M. A. Aksamit-Koperska, M. Sitarz, M. Missori, M. Krutzsch

**Affiliations:** 1 Jagiellonian University, Faculty of Chemistry, Ingardena 3, 30-060 Kraków, Poland; 2 BAM Federal Institute for Materials Research and Testing, Unter den Eichen 44-46, 12203, Berlin, Germany; 3 Jan Matejko Academy of Fine Arts, Faculty of Conservation and Restoration of Works of Art, Lea 27-29, 30-053 Kraków, Poland; 4 Faculty of Chemistry, Biological and Chemical Research Centre, University of Warsaw, Zwirki i Wigury 101, 02-089 Warsaw, Poland; 5 AGH University of Science and Technology, Faculty of Materials Science and Ceramics, Mickiewicza Av., 30-059 Cracow, Poland; 6 Consiglio Nazionale delle Ricerche, Institute for Complex Systems, Piazzale Aldo Moro 5, Rome, Italy; 7 Egyptian Museum and Papyrus Collection, Geschwister-Scholl-Str. 6, 10117 Berlin, Germany

## Abstract

Ancient papyri are a written heritage of culture that flourished more than 3000 years ago in Egypt. One of the most significant collections in the world is housed in the Egyptian Museum and Papyrus Collection in Berlin, from where the samples for our investigation come. The papyrologists, curators and conservators of such collections search intensely for the analytical detail that would allow ancient papyri to be distinguished from modern fabrications, in order to detect possible forgeries, assess papyrus deterioration state, and improve the design of storage conditions and conservation methods. This has become the aim of our investigation. The samples were studied by a number of methods, including spectroscopic (FTIR, fluorescent-FS, Raman) diffractional (XRD) and chromatographic (size exclusion chromatography-SEC), selected in order to determine degradation parameters: overall oxidation of lignocellulosic material, degree of polymerization and crystallinity of cellulose. The results were correlated with those obtained from carefully selected model samples including modern papyri and paper of different composition aged at elevated temperature in humid air. The methods were classified in the order SEC > FS > FTIR > XRD, based on their effectiveness in discriminating the state of papyri degradation. However, the most trustworthy evaluation of the age of papyri samples should rely on several methods.

Papyri served as a writing support for thousands of years. The first evidence for the use of papyrus in the production of writing substrates dates back to the third millennium BCE, in ancient Egypt. A very successful invention, papyrus spread well beyond Egypt’s borders and remained in use until the Middle Ages. Centuries after production, the ancient papyri were excavated (mainly in the beginning of the 20^th^ century, in Egyptian deserts) or purchased and then stored in several museums and libraries. Today, hundreds of thousands of extant papyrus fragments and rolls serve as an important source for historical, philosophical, linguistic and general cultural studies. For a considerable period of time, these studies were concerned mainly with the analysis of texts and the basic steps in the production of the individual papyrus sheets and rolls. The knowledge of these preparation steps was again based on texts: Pliny the Elder^1^ provided us with a general description of the manufacture of a papyrus writing surface.

On the other hand, the need for material preservation led to the appearance of an empirical body of knowledge collected by conservators^2^,^3^. These collected observations indicate that the manufacturing methods of papyrus sheets and rolls varied considerably depending on the time and place of production. It seems hardly surprising if we consider that papyrus was used for more than 4000 years, and over a wide geographical area. Recognizing the characteristic properties of each preparation type would allow one to date and localize the papyri of unknown provenance, and offer insight into trade routes. Correlating the texts with the quality of the writing support would also improve our understanding of the preparation of papyri for specific uses.

To this end, it is extremely important to distinguish the effect on a papyrus of natural ageing, compared to that caused by factors such as light, especially in the UV region of the spectrum, long exposure to high humidity, excessive heat, and short-term fluctuations of environmental conditions. Further factors such as wear, papyrus re-use in antiquity and historical conservation contribute considerably to the changes in the material state, as compared to pristine examples.

It is worth noting that the empirical data collected by conservators has not been matched by a systematic study of the physical and chemical properties of papyrus. In this work we have explored the potentiality of numerous methods including the vibrational spectroscopy, chromatography and X-ray diffraction for characterization of the natural degradation of papyrus. A particular question that we would like to address is whether any of the inherent properties of papyrus display ageing behaviour that can be scaled. In other words, is it possible to distinguish between ancient and modern papyri using conventional physicochemical techniques? For this purpose, Fourier Transform Infrared spectroscopy (FTIR), ultraviolet-visible (UV/VIS,) spectroscopy, UV-Vis fluorescence (FS) and Raman spectroscopy, X-ray diffraction (XRD) and size exclusion chromatography (SEC) were tested.

## State of the art

The literature concerning the physicochemical description of ancient papyri is not rich. The pioneering study using thermal analysis and pyrolysis mass spectrometry (MS) has shown that the ratio of cellulose and lignin, the main constituents of the papyrus writing material, vary with age^4^. The same authors reported later that, unfortunately, it depends also on the exact source of a papyrus, the manufacturing process, and degradation caused by the external factors^5^. The last conclusion was confirmed in the more recent studies of ancient and modern papyri, in which thermal analysis was accompanied by optical and electron microscopy with energy dispersed X-ray analysis or Particle Induced X-ray Emission (PIXE) analysis. Though the plant morphology could be observed despite the deformations induced by manufacturing processes, the cellulose-lignin ratios do not mirror the ageing process in a unique fashion^6^,^7^.

## Experimental

### Samples

The papyri samples used in this work comprise two modern papyri produced in Vienna and Berlin, and a sample of an ancient one from the Berlin collection (BL 111, excavated in February 1907 in Elephantine). The description is included in Table 1.

The reference samples were model papers provided by TNO, the Netherlands^8^. The composition and the properties of the samples are described in Table 1. The samples follow the trend of increasing complexity: from the high quality pure linter cellulose sample of P2, through the softwood bleached cellulose P1 (with traces of lignin), to the low quality acidic groundwood lignin-containing paper P3. The latter is supposed to mimic the composition of papyri which, according to the work by Wiedemann^4^, contain similar amounts of lignin. The reference paper samples were artificially aged in a climatic chamber at 90 °C, 59% RH in air for different periods up to 48 days, and then subjected to analysis.

## Methods

### Spectroscopic analysis

The *in situ* transmission FTIR measurements were performed in an Excalibur 3000 Diglab Spectrometer with DTGS detector. The spectrometer was equipped with a glass cuvette with ZnSe windows, an electrical furnace, and a gas supplying/evacuating system. The apparatus scheme is presented elsewhere^9^. Prior to analysis the samples, were thinned and placed in a cuvette equipped with a gas feeding and evacuating system, furnace and thermocouples. Then, the cuvette with the sample inside was evacuated and the temperature was raised to 70 °C, at which the sample was stored for 10 min to desorb water. The desorption progress was followed by the vanishing of the 1640 cm^−1^ band characteristic of bending vibrations of water molecules. Then the temperature was decreased to 30 °C and the spectra were collected for each sample.

For the sake of comparisons between various samples, the spectra were normalized using the internal standard method (integral of the band between 2800–3000 cm^−1^) described elsewhere^9^,^10^,^11^, and presented as standardized absorbance (A_std_). The degradation progress was traced in the range of 1500–1850 cm^−1^ where the carbonyl groups evolve. Quantitative analysis was carried out using the method described in our previous papers^9^,^11^ to obtain the standardized integral absorbance (in the range 1500–1850 cm^−1^ for P1 and P2, 1530–1850 cm^−1^ for P3 sample). The standardized integrals are thus a measure of the oxidation progress and the cellulose conversion towards carbonyl groups.

The Diffuse Reflectance Infrared Fourier Transform (DRIFT) spectra were collected by THERMO/Nicolet 5700 spectrometer with MCT/A detector equipped with a Harrick Praying Mantis appliance. A sample (diameter 5 mm) was placed inside the DRIFT appliance, which was continuously purged with dried helium (ca. 15 cm^3^/min). In order to remove water, the temperature of the appliance was set to 110 °C for ten seconds. Prior to measurements, the temperature was decreased to 30 °C, at which the spectra were collected.

The Fourier Transformed Raman (FT-Raman) accessory module coupled with a Nicolet 5700 FT-IR spectrometer was used for the FT-Raman measurements. The spectrometer was equipped with a 1064 nm Nd:YVO4 laser and CaF_2_ beam splitter. Raman spectra were collected using Raman backscattering collection geometry (180° configuration) and a germanium NXR Genie Raman detector. Each spectrum represents an average of 500 scans, obtained with a resolution 4 cm^−1^ in the range of Raman shift 100–3700 cm^−1^.

### Fluorescence analysis

Fluorescence (FS) mapping experiments were conducted with use of a Horiba Jobin Yvon FluoroLog-3 spectrofluorometer. The excitation range was between 300 to 1000 nm with a 10 nm increment, and emission was measured in the range of 300 to 1000 nm (even with a 10 nm increment). Entrance and exit slits were set in order to achieve a resolution of 5 nm. Integration time was equal to 0.1 s.

### SEC analysis

Cellulose SEC analysis was carried out using the derivatives of the original samples - cellulose tricarbanilate (CTC) - soluble in tetrahydrofuran (THF). The derivatives were prepared according to the procedure described by Stol and Lauriol^12^,^13^. Two CTC solutions were prepared for each paper sample and subjected to further analysis.

The average weighted molar mass (M_w_) and molar mass distributions were determined using a Waters chromatographic system, which consisted of: a Waters 1515 isocratic pump, a Waters 717+ autosampler, a column oven, a UV/VIS detector (set at the wavelength of 254 nm), a MALLS detector (Dawn Heleos, Wyatt Technology, working at 658 nm), and a differential refractive index (DRI) detector (Optilab t-rEX, Wyatt Technology, working at 658 nm). For the separation of the CTC samples, a set of two 25 × 1 cm mixed–bed polydivinylbenzene columns was used (Jordi). They were thermostated at 35 °C and proceeded by a guard column (Waters). THF (HPLC grade) was used as eluent with a flow rate of 1.0 cm^3^/min.

The SEC system was calibrated using 11 polystyrene standards of known molecular masses with narrow distributions (Fluka). The extinction coefficient for the UV/VIS detector was determined according to Lambert-Beer’s law, with the average value amounting to 7669 cm^3^/(g∙cm) (average from 23 measurements for P1 and P2 samples, standard deviation 11.2%). The specific refractive index increment (dn/dc) for the DRI detector was determined utilising the 100% recovery method delivered in ASTRA (Wyatt Technology) software. The average value of dn/dc for CTC from 10 measurements was 0.162 ml/g, standard deviation 0.016%^14^.

### Diffraction analysis

X-ray diffraction experiments were carried out with use of an X’Pert Pro MPD diffractometer (Phillips), equipped with a Johansson monochromator with copper Kα1 line (λ = 1,5405 Å) and a silicon, position sensitive X′Celerator detector. Measurements were performed in Bragg-Brentano θ–2θ geometry in the range of 2θ 10°–40°, increment of 0.008° and time of 240 seconds for each angular step. During measurements, a variable divergence slit was used to allow constant sensitivity to be obtained through whole range of 2 θ.

## Results

A vital problem associated with infrared spectroscopic analysis of cellulose containing ancient and contemporary materials is a band at around 1640 cm^−1^, which corresponds to bending vibrations of bound water present in cellulosic materials at ambient conditions. This band efficiently shields the frequency range where the vibrations of the products of cellulose degradation in the form of different carbonyl groups appear^9^,^11^,^15^. This practically prevents the analysis of the degradation products of all glucopyranosic polymers under ambient conditions. The remedy is to dispose of water from the studied material, either by evacuation or by increased temperature (T > 70 °C, for different temperatures, desorption time should be determined), which in practice means the use of a temperature cell where the samples are closed, evacuated and/or heated before they are subjected to analysis^9^,^10^.

**Figure 1:**
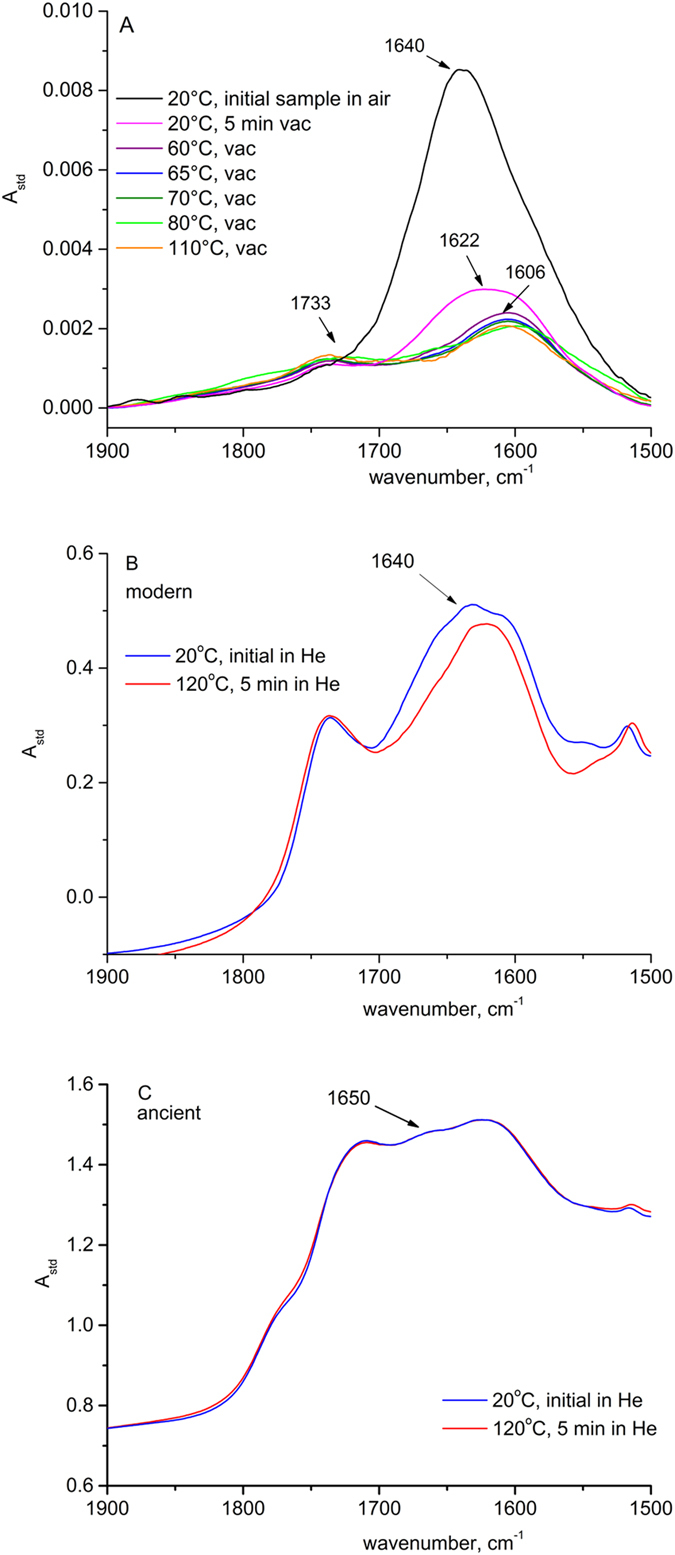
FTIR *in situ* analyses of bound water desorption from: (**A**) – P1 paper sample in vacuum at different temperatures (transmission measurements), DRIFT spectra collected at He flow at room temperature and at 120 °C after 5 min of conditioning: (**B**) – modern Berlin papyrus, (**C**) – ancient papyrus.

The effect of water desorption at elevated temperature on the FTIR spectra of reference paper and papyri, for both modern and ancient samples, can be seen in Fig. 1A–C. Figure 1A shows the transmission spectra of the P1 paper sample (Table 1) upon increasing temperature. Figure 1B and C presents the DRIFT spectra of the modern and ancient papyri samples, respectively, collected at room temperature and at 120 °C. In the first figure (A), the desorption of water from the P1 paper is manifested by the gradual disappearance of the 1640 cm^−1^ band until it is completely lost. In total, the drop is by around 0.005 in a A_std_ scale. A similar effect can be noted for both papyri samples (B and C). The modern papyrus sample (see Table 1 for description) shows the most significant decrease in standardized absorbance, reaching the value of 0.04 (B), while the value for the ancient papyrus reaches only 0.001 (C).

**Figure 2:**
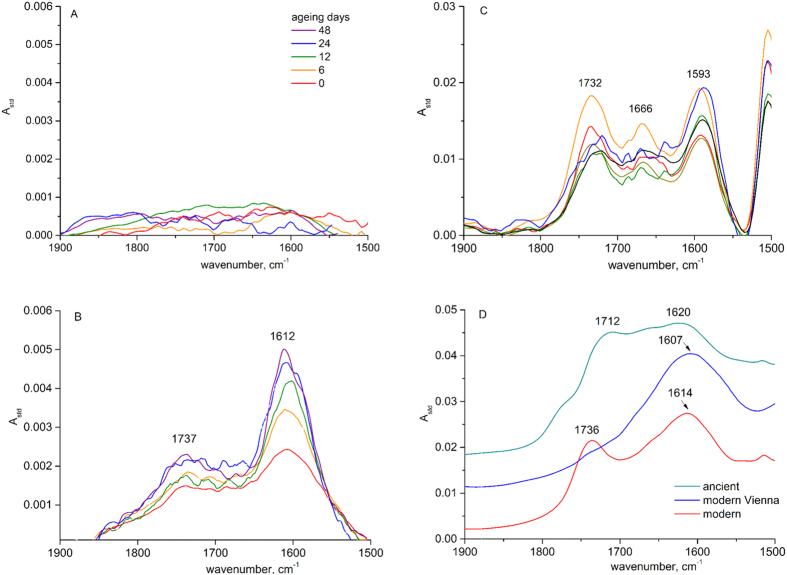
Comparison of transmission FTIR spectra of reference samples and DRIFT spectra of papyri: (collected in a temperature cell after water desorption at elevated temperature >70 °C); paper samples - initial and aged in climatic chamber for different periods: (**A**) – P2 (common legend to Fig. **A**–**C**), (**B**) – P1, (**C**) – P3; papyri samples: (**D**) – modern and ancient.

The effects of reference paper ageing on IR spectra are demonstrated in Fig. 2A–C. The spectral range was cut off to 1500–1900 cm^−1^ where we could expect the degradation products of cellulose and lignin in the form of different carbonyl groups. For the sake of both the semi-quantitative analysis of the carbonyl groups content and the comparison of degradation effects, the collected spectra were normalized using the internal standard method as described in the experimental section. The spectra of the initial samples (0 days of ageing) in Fig. 2 reflect their different compositions and the pre-treatment they were exposed to during production stage. The pure cellulose sample P2 does not, in its initial form, contain any carbonyl groups, while the P1 sample is slightly oxidized, as can be deduced from the two maxima at around 1737 and 1612 cm^−1^ assigned to aldehydic and conjugated ketonic groups, respectively, in our previous works^8^. The observed slight oxidation indubitably originates from paper bleaching. Although the initial spectrum of the lignin-containing sample P3 is much more difficult to interpret due to the vibrational pattern of benzene rings and –C=C bonds contained in the p-coumaryl, coniferyl and sinapyl alcohols building up the lignin polymer structure, three characteristic maxima can be distinguished, at 1732, 1666 and 1593 cm^−1^. The oxidation progress in different paper samples can be traced down by the evolution of carbonyl groups that emerge at different frequencies. The P2 paper sample containing pure cotton cellulose shows almost no oxidation effects upon ageing (Fig. 2A). The bleached softwood P1 sample oxidizes gradually with ageing time, as can be inferred from the profound increase in the intensity of the band at 1612 cm^−1^ (Fig. 2B) and the slight increase at 1737 cm^−1^. The first maximum was previously assigned to conjugated ketonic groups on C(2) and C(3) carbon atoms on glucopyranose rings, and the latter to the aldehydic groups^15^,^16^,^17^,^18^. There is no increasing tendency of intensities of the bands measured for the P3 lignin-rich sample with ageing time. However, it can be observed that the characteristic features of the vibrational pattern of lignin vanished after long exposure to air and high temperatures. This can be explained by the oxidation of alcohols, benzene rings and glucopyranose rings to many different carbonyl ensembles at different positions of the lignin and also cellulose polymers. A variety of carbonyl groups that emerged on the aged P3 sample is manifested in the spectra by multiple bands at different frequencies, that overlap one another and remaining lignin bands.

The IR (DRIFT) results collected for the papyri samples are presented in Fig. 2D. The spectra of two modern papyri samples are similar to that of the initial P3 samples, though the band at around 1666 cm^−1^ seems to be shielded by stronger bands, with the maximum at 1607 cm^−1^. The spectrum of the modern Vienna papyrus with only one broad maximum at 1607 cm^−1^ is different from other lignin-containing samples. The ancient papyrus, in turn, seems to resemble the oxidized lignocelluosic structure similar to that of the aged P3 (48 days, Fig. 2C).

**Figure 3:**
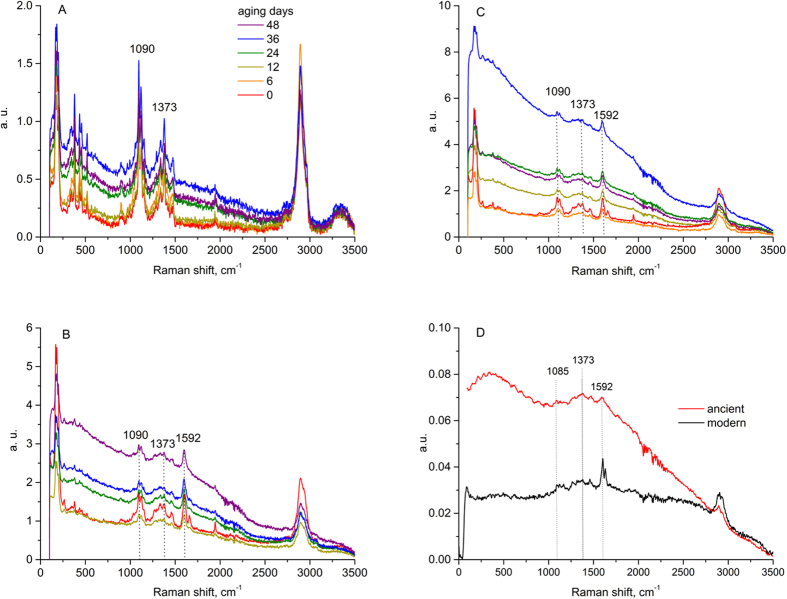
Comparison of FT-Raman spectra of the model paper samples aged in climatic chamber: (**A**) – P2 (common legend to Fig. **A**–**C**), (**B**) – P1 and (**C**) – P3 and papyri samples: (**D**) – modern and ancient.

The ageing impact on the same series of samples was also studied by FT Raman spectroscopy (Fig. 3A–D). Many vibrations coming from –C-H and –C=C groups are visible on the spectra of the initial paper samples. These were thoroughly assigned in previous papers by our group^9^,^11^. They are not discussed here, as the most profound effect arising from ageing of the paper samples was not the evolution of bands but the increasing fluorescence manifested by the rise of the background intensity of the spectra. This, in turn, resulted in the decrease in the intensities of the bands due to the decreased sensitivity of the analysis. This effect was found to depend on the lignin content in the sample, and on the ageing time. It is insignificant for the P2 paper, moderate for the P1 paper with low lignin content, and substantial for the P3 sample. The Raman spectra of the papyri samples show a similar trend in fluorescence with age: huge for the ancient papyri and moderate for the modern samples.

**Figure 4:**
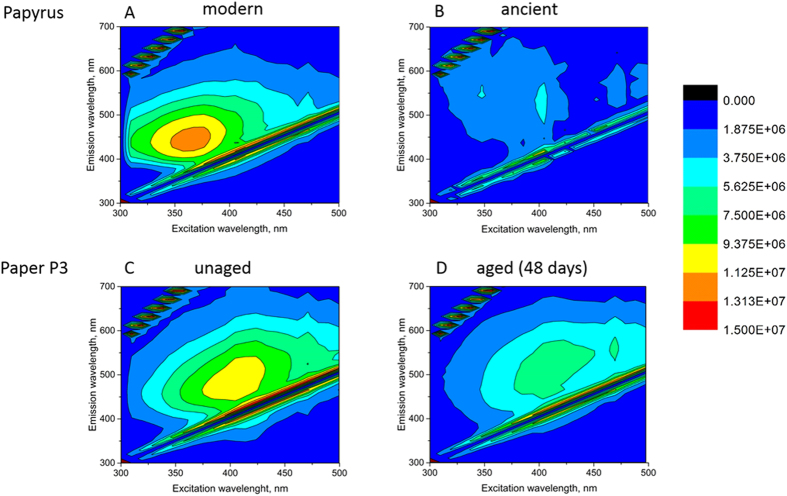
Fluorescence analysis of (**A**) – ancient and (**B**) – modern papyri samples as compared to the reference paper samples P3 (**C**) – initial and (**D**) – aged for 48 days.

This observation encouraged us to measure directly the fluorescence of the reference P3 and of the papyri samples. The results are shown in Fig. 4A–E, in form of fluorescence maps collected at the excitation range from near UV to mid-visible range (300–500 nm), and for the emitted signal in the same range. It is worth pointing out that the excitation range is different from that applied for the Raman analysis, as there an infrared laser was used (1064 nm). In the 300–500 nm range, the modern papyrus sample shows a single fluorescence maximum at around 430 nm (at 360 nm excitation), while the ancient sample displays almost no fluorescence signal. Similar behavior of decreasing fluorescence with the extent of degradation (oxidation) can be observed for P3: the initial P3 shows a maximum at around 490 nm (at 400 nm excitation), while the deeply aged P3 sample indicates no fluorescence.

**Figure 5:**
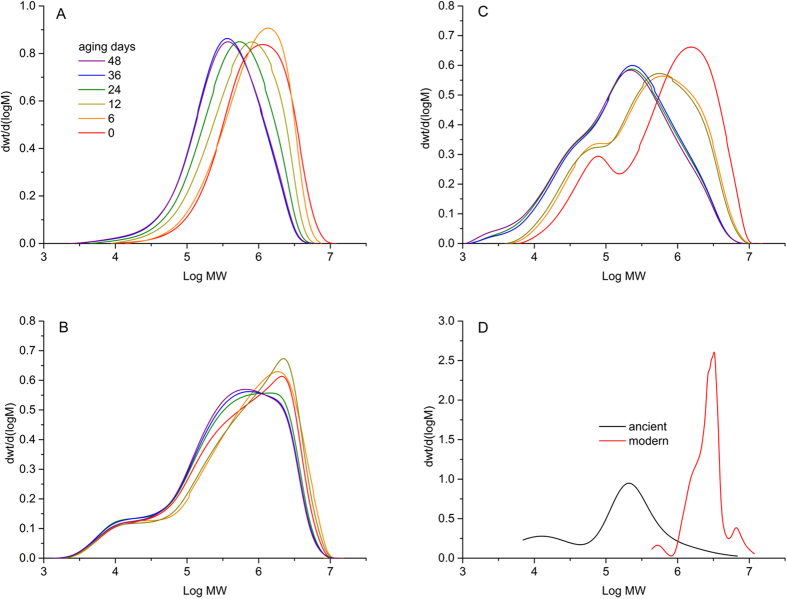
Comparison of molar mass distribution curves of the model paper samples aged in climatic chamber: (**A**) – P2 (common legend to Fig. **A**–**C**), (**B**) – P1 and (**C**) – P3 and papyri samples: (**D**) – modern and ancient.

The chromatographic SEC measurements were performed in order to determine the extent of depolymerization in the samples with ageing time. The curves representing the molar mass distribution of cellulose in the reference samples and the papyri samples are presented in Fig. 5A–D. The corresponding degree of polymerization (DP) values derived from the weight mean molar mass (M_w_) of cellulose according to the formula given in our previous works^8^,^14^,^19^,^20^ are presented in Table 2. It can be noted that the P2 and P3 initial samples have bimodal molar mass distribution due to the presence of hemicellulose that has (on average) lower mass than the mass of cellulose^8^,^19^. The general tendency observed for the reference samples is decreasing DP with ageing time. The extent of depolymerization expressed by the DP values follows the order: P2≪P1 < P3.

The mass distribution curve for the modern papyri sample shows modality similar to the P3 sample. The difference is the small fraction of high molar mass cellulose in the modern papyri. In the ancient papyri sample, the distribution is bimodal and the masses are shifted towards smaller values than those observed for the modern papyri sample. This is also reflected by the DP values (Table 2). There is a striking similarity in the position of maxima on the molar mass distribution curves between the pairs of samples: the P3 initial sample and the modern papyrus sample versus the P3 aged sample and the ancient papyrus.

**Figure 6:**
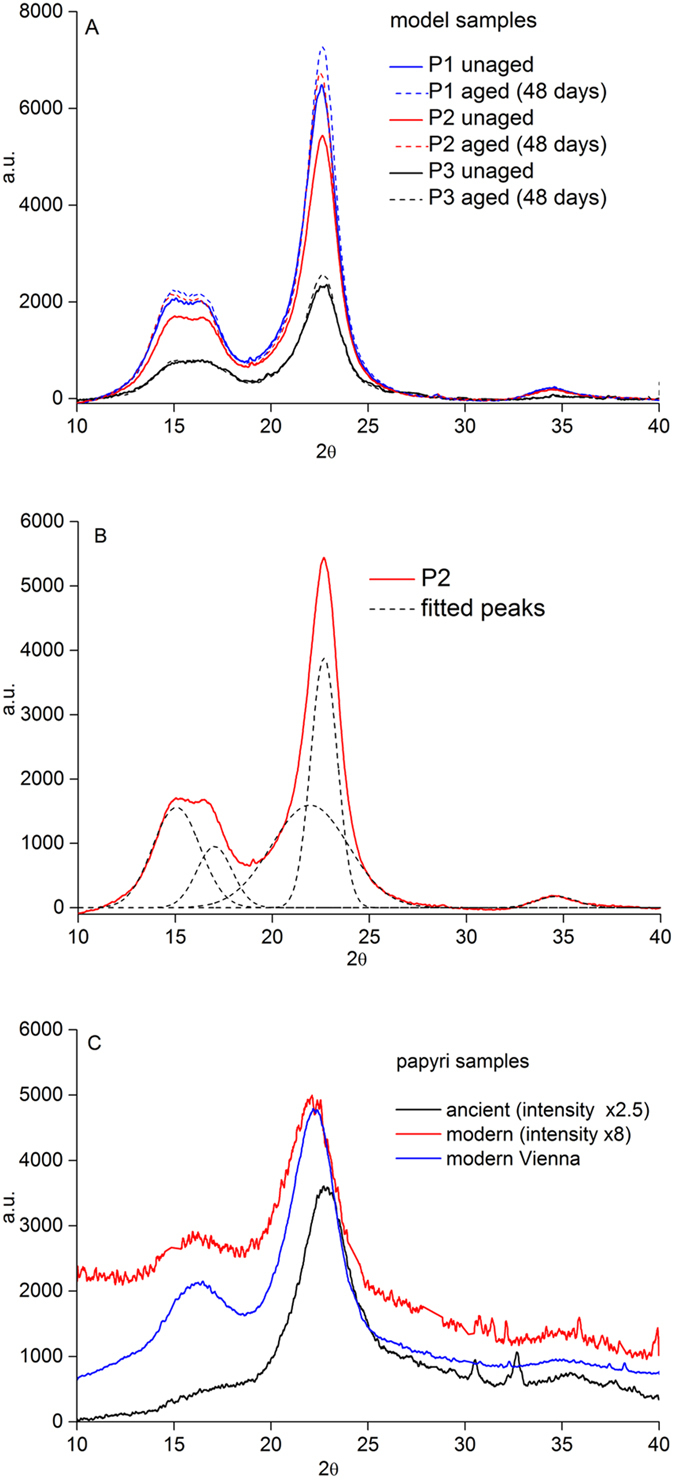
Comparison of the diffractograms obtained for (**A**) – model paper samples P1–P3 (unaged and aged for 48 days in climatic chamber), (**B**) – exemplary fitting results of a diffractogram of P2, (**C**) – modern and ancient papyri samples.

The crystallinity of the paper and papyri samples was also studied by XRD, the results of which are collected in Fig. 6A–C. Figure 6A presents the diffractograms obtained for the reference samples both unaged and aged for 48 days. Figure 6B shows an exemplary diffractogram of the unaged P2 sample with Gaussian curves fitted to it. The diffractograms of lignin-free papers consist of four peaks, which can be attributed to crystalline cellulose I_β_ - (101), (10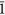), (002), (040) reflexes at 15.0°, 17.0°, 22.7° and 34.6° 2θ, respectively – and a wider peak at 21.9° attributed to amorphous cellulose^21^,^22^,^23^,^24^. The diffractograms of lignocellulosic materials (P3 and papyri samples) are similar, but this time the broader peak at 21.9° comprises the response of amorphous cellulose and lignin.

The crystallinity of the samples was estimated using two crystallinity indices (CI_dec_, CI_h_) calculated by methods described by Parks *et al*.^21^. CI_dec_ is defined as a percentage of areas of all crystalline reflexes in the total area under diffractogram. CI_h_ is defined as a contribution (percentage) of the difference in heights between (002) maximum and minimum at ca. 18.7° to the total height at (002) maximum (Fig. 6B). Table 3 summarizes the values of crystallinity indices calculated for both reference paper and papyri samples. CI_dec_ values provide information about the actual percentage of the cellulose crystalline phase in the studied material. The CI_h_ values do not have physical meaning, but can be used to indicate changes in samples’ crystallinity, or to compare samples in a series.

The results of XRD measurements obtained for the paper reference samples show that the lignin-free and low lignin content samples (P2, P1, respectively) possess quite similar crystallinity, with the indices higher than that of the lignin-rich sample P3. This can be accounted for by the amorphous structure of lignin, which occupies the same position on diffractograms as the amorphous cellulose. A comparison of crystallinity indices between unaged and aged model paper samples shows an expected rise of crystallinity with ageing time.

The XRD patterns of modern papyri samples shown in Fig. 6C bear only some resemblance to the difractograms of aged paper samples. Judging by the shape of the diffractograms, the tendency with age of the papyri samples is opposite to that of the reference aged paper samples. The crystallinity parameter CI_dec_ (Table 3) is much lower than that calculated for all unaged and aged paper samples, and there is no tendency at all with papyri age. The parameter CI_h_ seems more specific, as its value for the ancient papyrus is similar to that of the aged P3 sample.

## Discussion

What we know about the composition of papyri is that they contain 55–70% cellulose and 33–24% lignin, depending on their source, the remaining content being ash and bound water^4^. Our approach to understanding the structure of degraded ancient papyri samples is based on the adequate selection of reference samples of paper made of different kinds of plants and containing different amounts of lignin (Table 1), both pristine and aged at various conditions. The selection of the reference samples was made following the complexity of the material in the samples, where P2 represents high quality cotton cellulose while P3 mimics the composition of the papyri in terms of lignin content (compare data provided in Table 1). The P1 sample can thus be regarded as located in between the P2 and P3 sample with regard to the quality of fibers and lignin content. Thus, the reference samples should teach us about the ancient papyri samples through our observations of the changes induced by ageing.

**Figure 7:**
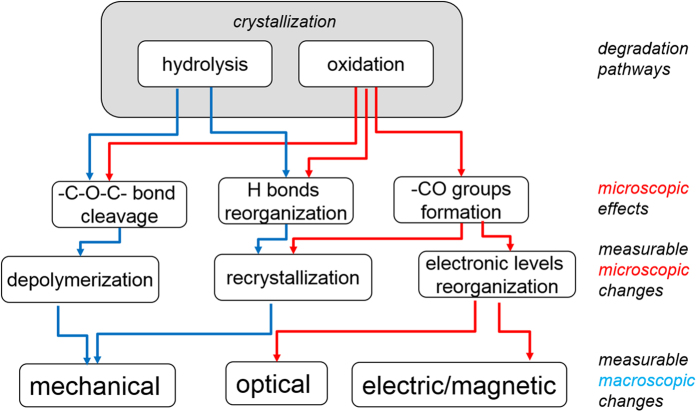
Schematics of the degradation pathways of lignocellulosic materials and their effect observable at different scales.

In general, the degradation of lignocellulosic systems proceeds along three reaction pathways: hydrolysis, oxidation and crystallization, which are interlaced with each other. The formation of carboxylic groups upon oxidation catalyzes the hydrolysis β,D-glycosidic bonds and, conversely, hydrolysis provides new reducing end groups for oxidation. What is more, hydrolysis always accompanies oxidation, since water emerges as a side product of oxidation. Taking into account that real paper samples contain already degraded cellulose and lignin, both of which are sources of the active oxygen species and radicals necessary to initiate oxidation, degradation seems self-sustainable once it starts. The general schematics of possible mechanisms of degradation, its effects and measurable observables is depicted in Fig. 7. Detailed information on degradation pathways can be found in our previous papers^8^,^25^.

An important general message to take is that recrystallization – the slowest structural change at ambient conditions - is a background against which the other, faster reactions proceed. Once the amorphous regions disappear in the material, the diffusion routes for water and oxygen are cut off and hydrolysis and oxidation cease. Thus we may expect that, for older samples, crystallized to a high degree, oxidation and hydrolysis is greatly suppressed and they do not age significantly any further unless exposed to a stimulus (such as temperature, humidity or light).

Oxidation initiated through the formation of hydroperoxides^26^ propagates through consecutive and parallel paths^9^,^15^ leading to the formation of various carbonyl groups, –C=O: i) starting from single ketones on C(2) and C(3) turning into conjugated diketones and ii) starting from aldehydes to carboxyles on C(6) carbon on glucopyranose rings. These can be measured by classic titrational methods^8^ or by FTIR and UV/Vis spectroscopic methods as presented by us before^10^,^27^.

From the infrared studies of the reference samples artificially degraded at elevated temperature we can learn that the effect of oxidation is different for different lignocellulosic materials, the most reluctant to deterioration being pure long chain cellulose from cotton in P2 samples (Fig. 2A). In materials that have already been slightly oxidized (P1) or contain high amounts of lignin (P3), oxidation proceeds faster (Fig. 2B and C). The oxidation products of the P1 sample were recognized as aldehydic groups at 1732 cm^−1^ and conjugated ketones at 1612 cm^−1^ 9,15. The oxidation products pattern is much harder to recognize in all lignin-containing samples (both aged P3 and papyri in Fig. 2C and D), due to the lignin vibrational pattern, which falls exactly into the range where the carbonyl groups occur. According to the literature, it can be expected that the oxidation proceeds first on lignin, which functions chemically as an antioxidant in plants^28^. Thus, we may assume that the lignin oxidizes first, and cellulose oxidation is supressed until lignin is fully oxidized. The oxidation of both lignin and cellulose results in a combination of many different carbonyl groups of different oxidation states (aldehydic, ketonic, carboxylic, chinone, etc.) and at different chemical environments. Thus, in the range 1600–1800 cm^−1^ the effect is a complex oxidation vibrational pattern composed of the multiple overlapping carbonyl bands and remaining lignin bands lacking distinct features that could be used to define oxidation gauges. An interesting observation is that the spectra in this range of both the ancient papyrus and the aged P3 sample resemble each other greatly, indicating the presence of various carbonyl ensembles. This, to some extent, can be used to evaluate the age of the samples, because FTIR has potential in recognizing old and modern papyri samples.

Although neither the extent of oxidation nor the changes in cellulose or lignin structure can be observed in Raman spectra, an interesting outcome of the scattered light analysis is the fluorescence changes. When the excitation source is near infrared (1064 nm) line, the fluorescence intensity increases with the increase of ageing time and lignin content, as observed for the reference samples. The effect is most profound for the lignin-containing P3 reference (Fig. 3A). For the excitation source ranging from UV to visible light, the effect is the opposite: fluorescence intensity decreases with age and lignin content in the samples. In both fluorescence regions (Raman spectra in Fig. 3 and fluorescence patterns in Fig. 4), the ancient papyri resemble the most aged sample P3. Thus, fluorescence can be used as a gauge to distinguish different papyri samples. Although thorough analysis of the excitation and de-excitation states in papyri would be difficult at this stage of our studies, a question that should be considered here is whether the fluorescence is a distinct feature of the aged lignocellulosic materials. An opposing argument to consider would be that perhaps fluorescence is characteristic of all ancient objects due to the presence of patina including dust, fat and all the pollutants the samples were exposed to throughout the ages. However, the latter possibility can be excluded since we observe the trend with fluorescence for clean, artificially aged paper reference samples.

Another property that can be used to describe the age of lignocellulosic materials is the degree of polymerization of cellulose. Depolymerization is an effect of both hydrolysis and oxidation (Fig. 7). While no comments are necessary regarding hydrolysis, oxidation- induced depolymerization is more complex. It acts through changing the conformation of glycopyranose rings (from a chair like structure towards flat rings, accompanied by a change in the sp^3^ to sp^2^ hybridization of carbon atoms), and through shifting the negative charge from oxygen atoms in the ring towards the oxygen atoms in the glycosidic bond. The latter effect has been demonstrated by DFT calculations of oxidized cellopentose^16^. No matter what the contribution of hydrolytic or oxidative glycosidic bond scissions, the overall depolymerization effects can be studied on the microscopic level by several methods, the most important and precise of which is SEC. SEC provides information on the whole distribution of the molecular masses of polymers^12^,^13^,^14^,^29^. When used with the multiple angle laser light scattering detector, it is claimed to be a direct method for absolute molar mass determination, or at least to be capable of approaching its real values^14^,^19^.

It does not seem astonishing that the ancient papyri samples have much lower DP than the modern counterparts, since depolymerization is a spontaneous process. The fact that the drop of DP of the aged P3 sample is similar to that of the ancient papyrus sample shows that we can spot the tendency in the DP values of lignocellulosic materials with their age. This tendency repeats for other physico-chemical properties of papyri presented above.

On the contrary, the diffractional results did not show clear tendency with the age of samples. The conclusions which can be drawn from XRD analyses are that, for the ancient papyri, the crystalline structure of the cellulose I_β_ seems to have vanished, unlike that in the P3 reference, which preserves the structure after many days of accelerated ageing. This seems reasonable, because structural reorganization of cellulose is a slow process that is evidently only slightly affected by temperature in the time frame studied by us. For this reason, artificial ageing by temperature does not seem to be an adequate method for mimicking the crystalline structure changes during natural ageing in real samples. The results also indicate that the XRD method is not able to reliably detect the changes induced in papyri by natural ageing, especially by the crystallinity indices described in the literature^21^,^24^.

The most important factor that affects the crystallinity of the samples seems to be the method of papyri manufacture, which involves many steps that probably include moistening and beating or pressing of the plant strips. Such treatment indubitably exerts an effect on the microscopic structure of cellulose fibers, which explains the different properties of the two modern samples observed both by XRD (Fig. 5C) and IR (Fig. 2D).

## Conclusions

The aim of this study was to provide analytical gauges to discriminate between ancient and modern papyri samples. The discriminative power of the methods which were used to evaluate the samples can be arranged in the following order: SEC > FS > FTIR > Raman > XRD. The classification is founded on the ability of a method to derive a single parameter able to distinguish between papyri samples of different ages.

The most efficient gauge is DP, which drops by almost 90% for ancient papyri samples with reference to the modern ones. The samples can also be easily distinguishable by the effect of FS excited at different ranges, even though the precise origin of this is not clearly understood. Since there are no single features in FTIR spectra that can be attributed to a specific change in the ancient papyri structure, the method does not seem to be sensitive enough to describe the oxidation progress in lignocellulosic materials. This is aside from the fact that it is possible to conclude in general that the ancient samples are more oxidized than the modern ones, which can be recognized by their “richer” carbonyl vibration pattern in the range of 1600–1800 cm^−1^. Regarding the high fluorescence of ancient samples, which was proved in this study to originate from oxidized lignin, the sensitivity of Raman analyses is low and hardly any Raman spectrum features can be distinguished. The fluorescence is indeed the prevailing signal that can be measured by Raman, even though analysis was performed at low power with long exposure times using the FT-Raman method with 1064 nm excitation laser, which is designed to supress fluorescence effects. The XRD is the least adequate for the assessment of the age of papyri samples, as the changes in crystallinity may also originate from mechanical processing of the samples at the preparation stage. This has been proved by comparing different modern papyri samples, which differ in crystallinity to a high extent.

Another important conclusion is that the most reliable evaluation of the age of papyri samples should rely on the use of the several analytical methods, preferably from the top of the list presented above. Analysis should be based on the comparison of different parameters derived from these methods for ancient and modern papyri samples, and preferably with reference to model paper samples composed of pure cellulose and lignin of the same concentration as in the studied papyri.

Another step in the evaluation of ancient papyri would be more precise determination of age. The methods presented here are able to distinguish between modern and ancient samples. However, we may imagine a situation in which the modern papyri samples were artificially aged for forgery purposes. As we can infer from the studies of the aged reference paper samples, the effect of artificial aging cannot be told apart from the effects of natural ageing by the methods used by us.

## Additional Information

**How to cite this article:** Łojewska, J. *et al*. Recognizing ancient papyri by a combination of spectroscopic, diffractional and chromatographic analytical tools. *Sci. Rep.* **7**, 46236; doi: 10.1038/srep46236 (2017).

**Publisher's note:** Springer Nature remains neutral with regard to jurisdictional claims in published maps and institutional affiliations.

## Figures and Tables

**Table 1:** Description of the reference paper samples and papyri samples.

Sample	Type	Lignin content	Ash conent wt%
P2	cotton linters cellulose	none	0.005
P1	softwood (bleached sulhpite) cellulose 99,5%	residual	0.45
P3	groundwood + softwood (bleached sulphite), sizing, filler	high (app. 30%)	20 (caolin, alum)
modern	produced in Berlin	high (app. 25%)	2
modern Vienna	produced in Vienna	high (app. 25%)	2
ancient	excavated in 1907 in Elephantine	high (app. 22–27%)	7–15

**Table 2:** Comparison of DP values of P3 reference paper sample (unaged and aged for 48 days in climatic chamber) and modern and ancient papyri samples.

Sample	DP	
	initial	aged (48 days)
P3	2100+/−95	1215+/−60
modern	5700+/−110	
ancient	800+/−70	

**Table 3:** Crystallinity indices, CI_dec_ (deconvolution) and CI_h_ (height), calculated for reference paper and papyri samples.

	CI_dec_		CI_h_	
	unaged	aged (48 days)	unaged	aged (48 days)
**reference paper samples**				
P2	63.9	66.3	88.1	90.4
P1	64.6	65.5	88.5	89.9
P3	60.7	61.8	84.7	86.9
**papyri samples**				
ancient	44.8		86.7	
modern	44.1		75.8	
modern Vienna	57.3		79.2	
